# Children’s measured exposure to food and beverage advertising on television in Toronto (Canada), May 2011–May 2019

**DOI:** 10.17269/s41997-021-00528-1

**Published:** 2021-06-15

**Authors:** Elise Pauzé, Monique Potvin Kent

**Affiliations:** 1 School of Epidemiology and Public Health, Faculty of Medicine, University of Ottawa, 600 Peter Morand Crescent, Ottawa, Ontario K1G 5Z3 Canada; 2 Interdisciplinary School of Health Sciences, Faculty of Health Sciences, University of Ottawa, Ottawa, ON Canada

**Keywords:** Public policy, Childhood obesity, Prevention, Food advertising, Politique publique, obésité infantile, prévention, publicités alimentaires

## Abstract

**Objective:**

Exposure to unhealthy food advertising is a known determinant of children’s poor dietary behaviours. The purpose of this study was to quantify and characterize Canadian children’s exposure to food advertising on broadcast television and examine trends over time.

**Methods:**

Objectively measured advertising exposure data for 19 food categories airing on 30 stations broadcast in Toronto were licenced for May 2011 and May 2019. Using ad ratings data, the average number of food advertisements viewed by children aged 2–11 years, overall, by food category and by type of television station (child-appealing, adolescent-appealing and generalist stations), was estimated per time period.

**Results:**

In May 2019, children viewed an average of 136 food advertisements on television, 20% fewer than in May 2011. More than half of advertisements viewed in May 2019 promoted unhealthy food categories such as fast food (43% of exposure), candy (6%), chocolate (6%) and regular soft drinks (5%) and only 17% of their total exposure occurred on child-appealing stations. Between May 2011 and May 2019, children’s exposure increased the most, in absolute terms, for savory snack foods (+7.2 ad exposures/child), fast food (+5.4) and regular soft drinks (+5.3) with most of these increases occurring on generalist stations.

**Conclusion:**

Canadian children are still exposed to advertisements promoting unhealthy food categories on television despite voluntary restrictions adopted by some food companies. Statutory restrictions should be adopted and designed such that children are effectively protected from unhealthy food advertising on both stations intended for general audiences and those appealing to younger audiences.

**Supplementary Information:**

The online version contains supplementary material available at 10.17269/s41997-021-00528-1.

## Résumé

**Objectif:**

Cette étude quantifiait et décrivait l’exposition des enfants canadiens à la publicité alimentaire télévisuelle et examinait l’évolution des tendances.

**Méthode:**

Des données sur l’exposition publicitaire pour 19 catégories alimentaires diffusées sur 30 postes de télévision à Toronto ont été obtenues sous licence pour les mois de mai 2011 et mai 2019. Le nombre moyen de publicités visionnées par enfant âgé de 2 à 11 ans, au total, par catégorie d’aliments et par type de poste (attrayants auprès d’enfants, d’adolescents ou d’un large public), a été estimé pour chaque période.

**Résultats:**

En mai 2019, les enfants ont visionné, en moyenne, 136 publicités alimentaires télévisuelles, soit 20 % de moins qu’en mai 2011. Plus de la moitié de leur exposition publicitaire en mai 2019 faisait la promotion de catégories d’aliments malsains tels que la restauration rapide (43 % de l’exposition), les bonbons (6 %), le chocolat (6 %) et les boissons gazeuses (5 %), et seulement 17 % de leur exposition totale est survenue sur les postes attrayants auprès d’enfants. De mai 2011 à mai 2019, l’exposition des enfants a augmenté le plus, en nombre absolu, pour les collations salées (+7,2 publicités/enfant), la restauration rapide (+5,4) et les boissons gazeuses (+5,3), la majorité de ces augmentations survenant sur les postes généralistes.

**Conclusion:**

Les enfants sont exposés à la publicité alimentaire télévisuelle malsaine malgré les restrictions volontaires adoptées par certaines compagnies alimentaires. Des lois protégeant adéquatement les enfants contre les publicités malsaines diffusées sur les postes généralistes et ceux attrayant aux jeunes devraient être envisagées.

## Introduction

In Canada, high body mass index and dietary risk factors are responsible for the second and fifth largest burden of disease in the country, respectively (Institute for Health Metrics and Evaluation, 2019). The nutritional health of Canadian children is of particular concern given that 31% of them have excess weight or obesity and most have diets inconsistent with national dietary guidelines, with ultra-processed foods which are typically high in sodium, fat and/or sugar accounting for more than half (52–57%) of their caloric intake on average (Jessri et al., 2016; Moubarac, 2017; Rao et al., 2016). As dietary behaviours are often established during childhood, this constitutes a critical period during which healthy dietary patterns should be promoted (Birch et al., 2007).

Food and beverage marketing, which overwhelmingly promotes energy-dense and nutrient-poor foods, has been established as an environmental determinant of children’s poor dietary behaviours (McGinnis et al., 2006; Norman et al., 2016; Sadeghirad et al., 2016). Several systematic reviews have concluded that food advertising influences the food purchases requested by children, their food preferences and their food intake during or immediately after exposure (McGinnis et al., 2006; Norman et al., 2016; Sadeghirad et al., 2016). Notably, a recent experimental study found that children do not compensate for the increased caloric intake that occurs after their exposure to food advertising at subsequent eating occasions, thereby strengthening the evidence of a causal link between exposure to food advertising and childhood obesity (Norman et al., 2018). Children’s heightened susceptibility to advertising stems in part from their inability to decipher commercial messages from other content before the age of 6 and to consistently understand advertising’s persuasive intentions by age 12 (Carter et al., 2011; Wilcox et al., 2004). As such, it is being increasingly argued that protecting children from unhealthy food advertising is necessary to uphold children’s right to health (Garde et al., 2018). There is in fact a global consensus that the marketing of foods high in sugar, fat and sodium to children should be restricted (World Health Organization, 2010).

Among Canadian children, television remains a significant source of exposure to advertising. In 2017/2018, children aged 2–11 years watched an average of 17.3 h of television per week according to measured media data, which exceeds current guidelines recommending recreational daily screen time be limited to 2 h or less among school-aged children (Canadian Radio-Television and Telecommunications Commission (CRTC), 2017; Canadian Society for Exercise Physiology, 2016). Currently, food advertising to children on television is mostly regulated by an industry-led voluntary initiative introduced in 2007 to which 16 companies, including McDonald’s, Kellogg’s and Hershey’s among others, currently participate (Ad Standards, 2019). Research however has shown that this initiative has been largely ineffective at improving the healthfulness of foods and beverages promoted to children on television (Potvin Kent & Wanless, 2014; Potvin Kent et al., 2011a; Kelly et al., 2019; Potvin Kent et al., 2018). For instance, a recent international study found that advertisements promoting unhealthy foods on three Canadian child speciality stations outnumbered those promoting healthier products by a factor of 12 and Canada had by far the highest rate of unhealthy food advertising (9.7 ads/h/station) compared with 21 other countries (the second highest being Spain at 5.2 ads/h/station) (Kelly et al., 2019). While most Canadian studies have focused on the frequency and/or healthfulness of food advertising on children’s speciality stations (Kelly et al., 2019), on child programming (Pinto et al., 2020) or on programs where children constitute a large proportion of viewers (Potvin Kent et al., 2018), comparatively fewer have examined children’s actual exposure using objectively measured media data (Potvin Kent & Wanless, 2014).

Recently, the federal government considered the Child Health Protection Act, a Bill that proposed restricting the advertising of foods and beverages high in sodium, saturated fat and sugar to children under 13 years in various media channels and settings (Senate of Canada, 2017; Health Canada, 2018). While this proposed Bill died on the order paper in June 2019, the current government has signaled its intention to introduce new restrictions to food marketing (Office of the Prime Minister, 2019). Considering these forthcoming restrictions, this study sought to provide critical baseline data needed to assess the impact of these restrictions and inform policy decisions regarding food advertising to children. Specifically, our objectives were to (1) determine the frequency of food and beverage advertising on broadcast television in a Canadian context, (2) estimate children’s exposure to this advertising using objectively measured exposure data, (3) examine children’s exposure by food category and type of station, and (4) describe changes in advertising frequency and exposure over time.

## Methods

Television audience measurement data were licenced from Numeris while television advertising data for 19 food categories broadcast on 31 to 35 television stations in Toronto were licenced from Nielsen Media Research for the month of May in 2011, 2013 and 2016 and from Numerator (formerly owned by Nielsen) for May 2019. Toronto was chosen because it is the largest Canadian broadcast market. The month of May was selected because it excludes any major holidays which may influence typical advertising spending and exposure. Four weeks of 24-h television programming for each period were included in the study (May 1 to May 28, 2011; April 28 to May 25, 2013; May 1 to May 28, 2016; and April 28 to May 25, 2019). These four years were selected as these data were previously licenced for other research (Potvin Kent et al., 2018). The 19 licenced food categories were cakes, candy, cold cereal, cheese, chocolate, compartment snacks, cookies, energy drinks, ice cream, juices, fruit nectars and drinks, frozen pizza, portable snacks, fast food restaurants, sit-down restaurants, snack foods, diet soft drinks, regular soft drinks, sports drinks and yogurt (see Supplemental Table 1 for a detailed description). These food categories were established by Nielsen/Numerator and were chosen because they constitute the most frequently advertised food categories to children (Kelly et al., 2019; Potvin Kent et al., 2011b).

The licenced data provide viewership information for all food advertisements that aired during the examined period. Audience viewership data are collected from a stratified probability sample of households that is proportional to the population. To capture viewership, data are collected on the time and station at which the television sets of households are tuned. Portable devices worn by each household member also record who is near the television when it is turned on. Being in the proximity of the television is therefore used as a proxy for viewership. To estimate audience viewership at the market level, data are weighted based on several characteristics, including age, sex, household size and type of television reception, among others. Ad viewership is then expressed as ‘rating points’ which is the estimated percentage of the population or selected age group who viewed the advertisement. To estimate the number of advertisements seen, on average, per child, the rating points for advertisements viewed by children aged 2 to 11 years were summed, overall and by food category, and then divided by 100. The sample of children on which exposure was estimated varied between May 2011 (*n* = 96), May 2013 (*n* = 187), May 2016 (*n* = 149) and May 2019 (*n* = 177). In May 2011, 2013 and 2016, rating points were reported by advertisement, whereas in May 2019, these were sometimes reported by advertised product. Data from May 2019 were therefore reviewed to remove duplicate advertisements and ensure data could be compared across time. In cases from May 2019 where multiple food categories were promoted within the same advertisement, the advertisement was classified into a single category in a manner consistent with data from previous years. For instance, ads promoting both cold cereal and portable snacks were classified under cold cereal exclusively while ads promoting both regular and diet soft drinks were classified under regular soft drinks.

In 2011, data from 34 stations were available; however, four were excluded from the study as Nielsen/Numerator stopped recording the advertisements on these stations before May 2019. The 30 stations included in the study were grouped into three station categories: child-appealing stations (*n* = 2), adolescent-appealing stations (*n* = 2) and generalist stations (*n* = 26). Teletoon and YTV were classified as child-appealing stations as most of their programs targeted children 12 years and younger. MTV and Much were deemed adolescent-appealing stations because much of their programming centered on either sitcoms, reality television and celebrity entertainment targeting or appealing to youth (i.e., MTV; e.g., Degrassi, Teen Mom, Ridiculousness, TMZ) or popular music and youth-appealing comedy shows and sitcoms (i.e., Much; e.g., Playlist, Tosh.O, South Park). A complete list of included stations is provided in Supplemental Table 2.

## Analysis

Nielsen’s Spotwatch software was used to extract television advertising data from May of 2011, 2013 and 2016, while Numerator’s Ad Quest software was used to extract data from May 2019. Datafiles were merged into one and analyses were conducted using SPSS version 27 (IBM 2020). The frequency of food advertising and children’s exposure on all stations was determined (overall and by food category) for each period. Food advertising frequency and exposure were tabulated by station category and described using frequencies. Absolute differences and percent changes in frequency and exposure between May 2011 and May 2019 were also calculated. For May 2019, the share of children’s exposure coming from child-appealing stations versus all other stations, overall and within food categories, was determined. Chi-square and post hoc *z*-tests with Bonferroni correction were performed to test differences in the distribution of ads by food category across examined periods, overall and by station category, when test assumptions were met. *p* values below 0.05 were considered statistically significant.

## Results

### Changes in food advertising across all stations

Overall, 80,018, 77,668, 84,361 and 102,409 food advertisements were broadcast on television in May of 2011, 2013, 2016 and 2019, respectively (Table 1). The distribution of ads by food category differed significantly across time (*χ*^2^ = 34,206.765; *df* = 54; *p* < 0.001). In May 2011, the most advertised food categories were fast food (33.0% of total ads), chocolate (11.4%) and yogurt (9.1%), while the most advertised food categories in May 2019 were fast food (44.2%), chocolate (8.3%) and cereal (8.3%). Between these two periods, the total frequency of ads increased by 28%; however, changes over time varied by food category. The largest relative increases were noted for cakes (+3392%) and regular soft drinks (+412%), while the largest relative decreases occurred for sports drinks (−95%) and portable snacks (−94%). In absolute terms, the greatest increases were noted for fast food (+18,907 ads), snack foods (+4937) and regular soft drinks (+4481), while the largest decreases were noted for yogurt (−4675), cookies (−2736) and portable snacks (−2246). The absolute and relative frequency of ads promoting snack foods and regular soft drinks increased steadily across all examined time periods while decreasing steadily for cookies, portable snacks and yogurt. For most other food categories, no consistent temporal trend was noted.

**Table 1:** Number of food and beverage advertisements broadcast on 30 television stations in Toronto (Canada) in May of 2011, 2013, 2016 and 2019, overall and by food category

Food/beverage category	May 2011, *n* (%)	May 2013, *n* (%)	May 2016, *n* (%)	May 2019, *n* (%)	% change, May 2011 to May 2019	Absolute difference, May 2019 vs May 2011
Cakes	52 (0.1)^a^	3 (<0.1)^a^	423 (0.5)^a^	1816 (1.8)^a^	+3392%	+1764
Candy	783 (1.0)^a^	4559 (5.9)^a^	3610 (4.3)^a^	3993 (3.9)^a^	+410%	+3210
Cereal	7074 (8.8)^a^	2970 (3.8)^a^	2915 (3.5)^a^	8454 (8.3)^a^	+20%	+1380
Cheese	5497 (6.9)^a^	3674 (4.7)^a^	4628 (5.5)^a^	3262 (3.2)^a^	−41%	−2235
Chocolate	9140 (11.4)^a,b^	10,922 (14.1)^a,b^	6771 (8.0)^a^	8463 (8.3)^b^	−7%	−677
Compartment snacks	0 (0)^a^	260 (0.3)^a,b,c^	0 (0)^b^	0 (0)^c^	-	0
Cookies	3765 (4.7)^a^	2210 (2.8)^a^	1387 (1.6)^a^	1029 (1.0)^a^	−73%	−2736
Energy drinks	765 (1.0)	804 (1.0)	857 (1.0)	957 (0.9)	+25%	+192
Ice cream	1254 (1.6)^a^	2136 (2.8)^a,b^	2612 (3.1)^a,b^	1574 (1.5)^b^	+26%	+320
Juices, drinks and nectars	3427 (4.3)^a,b^	4321 (5.6)^a^	4831 (5.7)^b^	3214 (3.1)^a,b^	−6%	−213
Pizza	2148 (2.7)^a^	1207 (1.6)^a^	1498 (1.8)^a^	1143 (1.1)^a^	−47%	−1005
Portable snacks	2397 (3.0)^a^	1892 (2.4)^a^	1739 (2.1)^a^	151 (0.1)^a^	−94%	−2246
Restaurant - fast food	26,370 (33.0)^a^	25,038 (32.2)^a^	34,041 (40.4)^a^	45,277 (44.2)^a^	+72%	+18,907
Restaurant - sit-down	5686 (7.1)^a^	4441 (5.7)^a,b^	5480 (6.5)^a,b^	7405 (7.2)^b^	+30%	+1719
Snack foods	2142 (2.7)^a,b^	4850 (6.2)^a,b^	5600 (6.6)^a^	7079 (6.9)^b^	+230%	+4937
Soft drinks - diet	804 (1.0)^a,b^	0 (0) ^a,b^	318 (0.4)^a^	413 (0.4)^b^	−49%	−391
Soft drinks - regular	1088 (1.4)^a,b^	3663 (4.7)^a^	3999 (4.7)^b^	5569 (5.4)^a,b^	+412%	+4481
Sports drinks	360 (0.4)^a^	83 (0.1)^a^	1006 (1.2)^a^	19 (<0.1)^a^	−95%	−341
Yogurt	7266 (9.1)^a^	4635 (6.0)^a^	2646 (3.1)^a^	2591 (2.5)^a^	−64%	−4675
Total	80,018 (100)	77,668 (100)	84,361 (100)	102,409 (100)	+28%	+22,391

The distribution of food ads by food category differs significantly across examined time periods (*χ*^2^ = 34206.765; *df* = 54; *p* < 0.001). Within rows, matching letters in superscript denote proportions that differ significantly (*p* < 0.05) according to post hoc *z*-tests with Bonferroni correction. Data source: Nielsen Media Research (2011, 2013, 2016) and Numerator (2019)

### Changes in food advertising by station category

On child-appealing stations, a total of 6173, 6274, 10,733 and 9832 food advertisements were broadcast in May of 2011, 2013, 2016 and 2019, respectively (Table 2). In May 2011, the most advertised food categories were fast food (33.0% of total ads), cereal (13.8%) and cheese (12.5%), while in May 2019, these were cold cereal (30.6%), fast food (21.8%) and candy (19.2%). Between May 2011 and May 2019, the total number of food advertisements on child-appealing stations increased by 59%. The greatest relative increases between these two periods were noted for ice cream (+2385%) and candy (+670%), while the largest relative decreases were noted for cookies (−100%), pizza (−100%) and regular soft drinks (−100%). The largest absolute increases between these two periods occurred for cereal (+2156 ads) and candy (+1641), while the largest absolute decreases were noted for portable snacks (−383) and cookies (−359). For most food categories, there was no consistent temporal trend in advertising frequency across all examined periods. Only the number of cookie advertisements decreased steadily over time. Diet soft drinks and sports drinks were not advertised at all on child-appealing stations across all examined periods.

**Table 2:** Number of food and beverage advertisements airing on television in Toronto (Canada) in May 2011, 2013, 2016 and 2019, by food category and by television station type

	Child-appealing stations (*n* = 2)					Adolescent-appealing stations (*n* = 2)					Generalist stations (*n* = 26)				
Food/beverage category	May 2011 *n* (%)	May 2013 *n* (%)	May 2016 *n* (%)	May 2019 *n* (%)	% change, May 2011 to May 2019	May 2011 *n* (%)	May 2013 *n* (%)	May 2016 *n* (%)	May 2019 *n* (%)	% change, May 2011 to May 2019	May 2011 *n* (%)	May 2013 *n* (%)	May 2016 *n* (%)	May 2019 *n* (%)	% change, May 2011 to May 2019
Cakes	0 (0)	0 (0)	0 (0)	294 (3.0)	-	0 (0)^a^	0 (0)^b^	0 (0)^c^	312 (2.8)^a,b,c^	-	52 (0.1)^d^	3 (<0.1)^d^	423 (0.6)^d^	1210 (1.5)^d^	+2227%
Candy	245 (4.0)	877 (14.0)	2041 (19.0)	1886 (19.2)	+670%	84 (1.9)^a,b^	923 (13.3)^a,b^	54 (0.9)^a^	72 (0.6)^b^	− 14%	454 (0.7)^d^	2759 (4.3)^d^	1515 (2.2)^d^	2035 (2.5)^d^	+348%
Cereal	853 (13.8)	401 (6.4)	1224 (11.4)	3009 (30.6)	+253%	0 (0)^a^	0 (0)^b^	0 (0)^c^	159 (1.4)^a,b,c^	-	6221 (9.0)^d^	2569 (4.0)^d^	1691 (2.5)^d^	5286 (6.5)^d^	− 15%
Cheese	774 (12.5)	505 (8.0)	1122 (10.5)	416 (4.2)	− 46%	252 (5.6)^a,b^	0 (0)^a^	0 (0)^b^	142 (1.3)^a,b^	− 44%	4471 (6.5)^d,e^	3169 (4.9)^d^	3506 (5.2)^e^	2704 (3.3)^d,e^	− 40%
Chocolate	218 (3.5)	691 (11.0)	789 (7.4)	214 (2.2)	− 2%	658 (14.5)^a^	387 (5.6)^a^	1172 (19.7)^a^	1015 (9.2)^a^	+54%	8264 (11.9)^d^	9844 (15.3)^d^	4810 (7.1)^d^	7234 (8.9)^d^	− 12%
Compartment snacks	0 (0)	3 (< 0.1)	0 (0)	0 (0)	-	0 (0)	0 (0)	0 (0)	0 (0)	-	0 (0)^d^	257 (0.4)^d,e,f^	0 (0)^e^	0 (0)^f^	-
Cookies	359 (5.8)	155 (2.5)	48 (0.4)	0 (0)	− 100%	465 (10.3)^a^	215 (3.1)^a^	0 (0)^a^	40 (0.4)^a^	− 91%	2941 (4.2)^d^	1840 (2.9)^d^	1339 (2.0)^d^	989 (1.2)^d^	− 66%
Energy drinks	0 (0)	55 (0.9)	85 (0.8)	61 (0.6)	-	233 (5.1)^a^	257 (3.7)^a,b^	316 (5.3)^b^	216 (1.9)^a,b^	− 7%	532 (0.8)	492 (0.8)	456 (0.7)^d^	680 (0.8)^d^	+28%
Ice cream	27 (0.4)	503 (8.0)	199 (1.9)	671 (6.8)	+2385%	0 (0)	0 (0)	0 (0)	0 (0)	-	1227 (1.8)^d^	1633 (2.5)^d^	2413 (3.6)^d^	903 (1.1)^d^	− 26%
Juices, drinks and nectars	200 (3.2)	105 (1.7)	375 (3.5)	85 (0.9)	− 58%	180 (4.0)^a^	794 (11.4)^a^	0 (0)^a^	316 (2.9)^a^	+76%	3047 (4.4)^d^	3422 (5.3)^d^	4456 (6.6)^d^	2813 (3.5)^d^	− 8%
Pizza	125 (2.0)	0 (0)	191 (1.8)	0 (0)	− 100%	0 (0)^a^	0 (0)^b^	0 (0)^c^	209 (1.9)^a,b,c^	-	2023 (2.9)^d,e^	1207 (1.9)^d^	1307 (1.9)^e^	934 (1.1)^d,e^	− 54%
Portable snacks	443 (7.2)	173 (2.8)	421 (3.9)	60 (0.6)	− 86%	0 (0)	0 (0)	0 (0)	0 (0)	-	1954 (2.8)^d^	1719 (2.7)^e^	1318 (1.9)^d,e^	91 (0.1)^d,e^	− 95%
Restaurant - fast food	2037 (33.0)	2156 (34.4)	3083 (28.7)	2140 (21.8)	+5%	1465 (32.3)^a^	1125 (16.1)^a^	2230 (37.4)^a^	6562 (59.2)^a^	+348%	22,868 (33.0)^d^	21,757 (33.8)^d^	28,728 (42.5)^d^	36,575 (44.9)^d^	+60%
Restaurant - sit-down	137 (2.2)	256 (4.1)	89 (0.8)	432 (4.4)	+215%	120 (2.6)^a^	169 (2.4)^b^	323 (5.4)^a,b,c^	227 (2.0)^c^	+89%	5429 (7.8)^d^	4016 (6.2)^d,e^	5068 (7.5)^e^	6746 (8.3)^d,e^	+24%
Snack foods	30 (0.5)	0 (0)	319 (3.0)	208 (2.1)	+593%	224 (4.9)^a,b^	503 (7.2)^a^	722 (12.1)^a,b^	777 (7.0)^b^	+247%	1888 (2.7)^d,e^	4347 (6.7)^d^	4559 (6.7)^e^	6094 (7.5)^d,e^	+223%
Soft drinks - diet	0 (0)	0 (0)	0 (0)	0 (0)	-	0 (0)^a^	0 (0)^b^	0 (0)^c^	101 (0.9)^a,b,c^	-	804 (1.2)^d,e^	0 (0)^d,e^	318 (0.5)^d^	312 (0.4)^e^	− 61%
Soft drinks - regular	78 (1.3)	156 (2.5)	255 (2.4)	0 (0)	− 100%	367 (8.1)^a^	1922 (27.6)^a,b^	934 (15.7)^a,b^	887 (8.0)^b^	+142%	643 (0.9)^d^	1585 (2.5)^d^	2810 (4.2)^d^	4682 (5.7)^d^	+628%
Sports drinks	0 (0)	0 (0)	0 (0)	0 (0)		234 (5.2)^a,b^	0 (0)^a^	78 (1.3)^a,b^	0 (0)^b^	− 100%	126 (0.2)^d^	83 (0.1)^e^	928 (1.4)^d,e^	19 (<0.1)^d,e^	− 85%
Yogurt	647 (10.5)	238 (3.8)	492 (4.6)	356 (3.6)	− 45%	248 (5.5)^a^	671 (9.6)^a^	135 (2.3)^a^	50 (0.5)^a^	− 80%	6371 (9.2)^d^	3726 (5.8)^d^	2019 (3.0)^d^	2185 (2.7)^d^	− 66%
Total	6173 (100)	6274 (100)	10,733 (100)	9832 (100)	+59%	4530 (100)	6966 (100)	5964 (100)	11,085 (100)	+145%	69,315 (100)	64,428 (100)	67,664 (100)	81,492 (100)	+18%

Child-appealing stations included Teletoon and YTV. Adolescent-appealing stations included Much and MTV. All other stations were classified as generalist stations. The distribution of food ads by food category differs significantly across examined time periods on adolescent-appealing (*χ*^2^ = 12,962.931; *df* = 45; *p* < 0.001) and generalist (*χ*^2^ = 30,289.205; *df* = 54; *p* < 0.001) stations. This could not be tested on child-appealing stations as test assumptions were not met. Within rows, matching letters in superscript denote proportions that differ significantly (*p* < 0.05) according to post hoc *z*-tests with Bonferroni correction. Data source: Nielsen Media Research (2011, 2013, 2016) and Numerator (2019).

Overall, a total of 4530, 6966, 5964 and 11,085 food advertisements were broadcast on adolescent-appealing stations in May of 2011, 2013, 2016 and 2019, respectively. On these stations, the distribution of ads by food category differed significantly across time (*χ*^2^ = 12,962.931; *df* = 45; *p* < 0.001). The most advertised food categories in May 2011 were fast food (32.3% of total ads), chocolate (14.5%) and cookies (10.3%), while in May 2019, these were fast food (59.2%), chocolate (9.2%) and regular soft drinks (8.0%). Between May 2011 and May 2019, the total number of food advertisements on adolescent-appealing stations increased by 145%. The greatest relative increases between these two periods occurred for fast food (+348%) and snack foods (+247%), while the largest relative decreases were noted for sports drinks (−100%) and cookies (−91%). The largest absolute increases between these two periods were noted for fast food (+5097 ads), snack foods (+553) and regular soft drinks (+520), while the largest absolute decreases were noted for cookies (−425) and sports drinks (−234). For most food categories, there was no consistent temporal trend in advertising frequency across all examined periods. Only the number of snack food advertisements increased steadily over time. Ice cream and portable and compartment snacks were not at all advertised on adolescent-appealing stations across all examined periods.

On generalist stations, a total of 69,315, 64,428, 67,664 and 81,492 food advertisements aired in May of 2011, 2013, 2016 and 2019, respectively. On these stations, the distribution of ads by food category differed significantly across time (*χ*^2^ = 30,289.205; *df* = 54; *p* < 0.001). The most advertised food categories in May 2011 were fast food (33.0% of total ads), chocolate (11.9%) and yogurt (9.2%), while in May 2019, these were fast food (44.9%), chocolate (8.9%) and sit-down restaurants (8.3%). Between May 2011 and May 2019, the number of food advertisements on generalist stations increased by 18%. The greatest relative increases between these two periods occurred for cakes (+2227%) and regular soft drinks (+628%), while the largest relative decreases were noted for portable snacks (−95%) and sports drinks (−85%). The largest absolute increases between these two periods were noted for fast food (+13,707 ads), snack foods (+4206) and regular soft drinks (+4039), while the largest absolute decreases were noted for yogurt (−4186), cookies (−1952) and portable snacks (−1863). The absolute and relative frequency of ads promoting snack foods and regular soft drinks increased steadily across all examined periods while decreasing steadily for cookies and portable snacks. No other consistent temporal trends among food categories were noted.

### Changes in children’s food advertising exposure, overall and by station category

On average, children were exposed to 170, 150, 162 and 136 food advertisements in May of 2011, 2013, 2016 and 2019, respectively (Table 3). The food categories to which children were the most exposed in May 2011 were fast food (31.3% of total exposure), cereal (13.6%) and yogurt (9.4%), while in May 2019, these were fast food (43.0%), cereal (10.9%) and sit-down restaurants (8.8%). Children’s overall exposure to food advertising decreased by 20% or 33.8 ads per child between May 2011 and May 2019. While exposure decreased greatly in relative terms for some food categories, such as portable snacks (−97%) and cookies (−89%), others, such as regular soft drinks (+495%), cakes (+481%) and snack foods (+279%), increased markedly. In absolute terms, children’s exposure increased the most between May 2011 and May 2019 for snack foods (+7.2 ads/child), fast food (+5.4) and regular soft drinks (+5.3), while decreasing the most for yogurt (−12.7 ads/child), cheese (−10.2) and portable snacks (−10.2). Children’s exposure in both relative and absolute terms decreased steadily across all examined periods for cookies, pizza and yogurt while increasing steadily for snack foods and regular soft drinks.

**Table 3:** Children’s exposure to food and beverage advertisements on 30 stations broadcast in Toronto (Canada) in May of 2011, 2013, 2016 and 2019, by food category

Food/beverage category	Average number of advertisements viewed by children (aged 2–11 years)					
	May 2011, *n* (%)	May 2013, *n* (%)	May 2016, *n* (%)	May 2019, *n* (%)	% change, May 2011 to May 2019	Absolute difference, May 2019 vs May 2011
Cakes	0.27 (0.2)	0 (0)	0.69 (0.4)	1.57 (1.2)	+481%	+1.3
Candy	4.18 (2.5)	11 (7.3)	13.03 (8.1)	7.74 (5.7)	+85%	+3.6
Cereal	23.23 (13.6)	9.11 (6.1)	12.59 (7.8)	14.81(10.9)	−36%	−8.4
Cheese	14.18 (8.3)	11.05 (7.3)	12.27 (7.6)	4.02 (2.9)	−72%	−10.2
Chocolate	12.71 (7.5)	14.64 (9.7)	7.85 (4.9)	8.57 (6.3)	−33%	−4.1
Compartment snacks	0 (0)	0.31 (0.2)	0 (0)	0 (0)	-	0.0
Cookies	6.18 (3.6)	3.86 (2.6)	2.24 (1.4)	0.7 (0.5)	−89%	−5.5
Energy drinks	0.96 (0.6)	0.88 (0.6)	1.24 (0.8)	1.35 (1.0)	+41%	+0.4
Ice cream	2.31 (1.4)	3.5 (2.3)	4.42 (2.7)	1.09 (0.8)	−53%	−1.2
Juices, drinks and nectars	7.32 (4.3)	8.03 (5.3)	7.93 (4.9)	3.68 (2.7)	−50%	−3.6
Pizza	4.85 (2.8)	2.12 (1.4)	2.05 (1.3)	1.08 (0.8)	−78%	−3.8
Portable snacks	10.48 (6.2)	4.53 (3.0)	5.05 (3.1)	0.27 (0.2)	−97%	−10.2
Restaurant - fast food	53.29 (31.3)	52.99 (35.2)	64.45 (39.9)	58.68 (43.0)	+10%	+5.4
Restaurant - sit-down	9.03 (5.3)	9.87 (6.6)	7.4 (4.6)	11.94 (8.8)	+32%	+2.9
Snack foods	2.59 (1.5)	5.09 (3.4)	6.99 (4.3)	9.81 (7.2)	+279%	+7.2
Soft drinks - diet	1.1 (0.6)	-	0.66 (0.4)	0.54 (0.4)	−51%	−0.6
Soft drinks - regular	1.06 (0.6)	3.62 (2.4)	5.5 (3.4)	6.31 (4.6)	+495%	+5.3
Sports drinks	0.55 (0.3)	0.31 (0.2)	1.57 (1.0)	1.04 (0.8)	+89%	+0.5
Yogurt	15.93 (9.4)	9.57 (6.4)	5.65 (3.5)	3.19 (2.3)	−80%	−12.7
Total	170.2 (100)	150.5 (100)	161.6 (100)	136.4 (100)	−20%	−33.8

Data source: Nielsen Media Research (2011, 2013, 2016) and Numerator (2019).

As shown in Table 4, changes in children’s overall exposure to food advertising also differed by station category. Children’s total exposure decreased between May 2011 and May 2019 by 59% (−33.5 ads/child) and 3% (−3.5 ads/child) on child-appealing and generalist stations, respectively, while increasing by 237% (+3.1 ads/child) on adolescent-appealing stations. Children’s exposure to fast food and regular soft drink advertising decreased on child-appealing stations (−68% or −10.8 ads/child and −100% or −0.3 ads/child, respectively) but increased on adolescent-appealing (+600% or +2.2 ads/child and +50% or +0.1 ad/child, respectively) and generalist stations (+38% or +14.0 ads/child and +884% or +5.5 ads/child, respectively). For other food categories such as snack foods and sit-down restaurants, greater absolute increases in children’s exposure also occurred on generalist stations (+7.0 and +2.6 ads/child, respectively) compared with child-appealing stations (+0.02 and +0.2 ads/child, respectively).

**Table 4:** Children’s exposure to food and beverage advertisements broadcast in Toronto (Canada) in May 2011, 2013, 2016 and 2019, by food category and television station type

Food/beverage category	Average number of advertisements viewed by children (aged 2–11 years)														
	Child-appealing stations (*n* = 2)					Adolescent-appealing stations (*n* = 2)					Generalist stations (*n* = 26)				
	May 2011 *n* (%)	May 2013 *n* (%)	May 2016 *n* (%)	May 2019 *n* (%)	% change, May 2011 to May 2019	May 2011 *n* (%)	May 2013 *n* (%)	May 2016 *n* (%)	May 2019 *n* (%)	% change, May 2011 to May 2019	May 2011 *n* (%)	May 2013 *n* (%)	May 2016 *n* (%)	May 2019 *n* (%)	% change, May 2011 to May 2019
Cakes	0 (0)	0 (0)	0 (0)	0.1 (0.4)	-	0 (0)	0 (0)	0 (0)	0.03 (0.7)	-	0.27 (0.2)	0 (0)	0.69 (0.6)	1.43 (1.3)	+430%
Candy	3.70 (6.5)	7.65 (18.6)	10.97 (21.1)	5.85 (24.8)	+58%	0.03 (2.3)	0.24 (13.7)	0 (0)	0.02 (0.4)	− 33%	0.44 (0.4)	3.10 (2.9)	2.06 (1.9)	1.87 (1.7)	+325%
Cereal	12.96 (22.7)	5.38 (13.1)	9.41 (18.1)	8.24 (34.9)	− 36%	0 (0)	0 (0)	0 (0)	0.22 (4.9)	-	10.27 (9.2)	3.73 (3.5)	3.18 (2.9)	6.35 (5.9)	− 38%
Cheese	6.50 (11.4)	5.08 (12.4)	5.40 (10.4)	1.42 (6.0)	− 78%	0.03 (2.3)	0 (0)	0 (0)	0.04 (0.9)	33%	7.65 (6.8)	5.97 (5.5)	6.87 (6.4)	2.56 (2.4)	− 67%
Chocolate	0.54 (0.9)	1.41 (3.4)	2.55 (4.9)	0.33 (1.4)	− 39%	0.16 (12.1)	0.05 (2.9)	0.12 (7.7)	0.50 (11.2)	213%	12.01 (10.7)	13.17 (12.2)	5.18 (4.8)	7.74 (7.1)	− 36%
Compartment snacks	0 (0)	0 (0)	0 (0)	0 (0)	-	0 (0)	0 (0)	0 (0)	0 (0)	-	0 (0)	0.31 (0.3)	0 (0)	0 (0)	-
Cookies	1.45 (2.5)	0.81 (2.0)	0.19 (0.4)	0 (0)	− 100%	0.23 (17.4)	0.08 (4.6)	0 (0)	0.04 (0.9)	− 83%	4.49 (4.0)	2.97 (2.8)	2.05 (1.9)	0.66 (0.6)	− 85%
Energy drinks	0 (0)	0.07 (0.2)	0.28 (0.5)	0.02 (0.1)	-	0.07 (5.3)	0.04 (2.3)	0.05 (3.2)	0.14 (3.1)	+100%	0.89 (0.8)	0.77 (0.7)	0.92 (0.9)	1.19 (1.1)	+34%
Ice cream	0.46 (0.8)	1.69 (4.1)	1.11 (2.1)	0.32 (1.4)	− 30%	0 (0)	0 (0)	0 (0)	0 (0)	-	1.85 (1.7)	1.81 (1.7)	3.30 (3.1)	0.77 (0.7)	− 58%
Juices, drinks and nectars	1.25 (2.2)	0.85 (2.1)	1.53 (2.9)	0.02 (0.1)	− 98%	0.06 (4.5)	0.28 (16.0)	0 (0)	0.11 (2.5)	+83%	6.00 (5.4)	6.90 (6.4)	6.40 (5.9)	3.56 (3.3)	− 41%
Pizza	0.22 (0.4)	0 (0)	0.74 (1.4)	0 (0)	− 100%	0 (0)	0 (0)	0 (0)	0.14 (3.1)	-	4.63 (4.1)	2.12 (2.0)	1.31 (1.2)	0.94 (0.9)	− 80%
Portable snacks	6.87 (12.0)	2.20 (5.3)	3.30 (6.3)	0.16 (0.7)	− 98%	0 (0)	0 (0)	0 (0)	0 (0)	-	3.61 (3.2)	2.33 (2.2)	1.75 (1.6)	0.11 (0.1)	− 97%
Restaurant - fast food	15.81 (27.7)	10.68 (26.0)	12.09 (23.2)	5.00 (21.2)	− 68%	0.37 (28.0)	0.28 (16.0)	0.75 (48.1)	2.59 (58.2)	+600%	37.11 (33.2)	42.03 (39.1)	51.60 (47.8)	51.09 (47.2)	+38%
Restaurant - sit-down	0.63 (1.1)	1.56 (3.8)	0.35 (0.7)	0.80 (3.4)	+27%	0.04 (3.0)	0.08 (4.6)	0.06 (3.8)	0.14 (3.1)	+250%	8.36 (7.5)	8.24 (7.7)	6.99 (6.5)	11.00 (10.2)	+32%
Snack foods	0.26 (0.5)	0 (0)	0.63 (1.2)	0.28 (1.2)	+8%	0.08 (6.1)	0.13 (7.4)	0.18 (11.5)	0.25 (5.6)	+213%	2.25 (2.0)	4.96 (4.6)	6.19 (5.7)	9.28 (8.6)	+312%
Soft drinks - diet	0 (0)	0 (0)	0 (0)	0 (0)	-	0 (0)	0 (0)	0 (0)	0.02 (0.4)	-	1.10 (1.0)	0 (0)	0.66 (0.6)	0.52 (0.5)	− 53%
Soft drinks - regular	0.31 (0.5)	1.02 (2.5)	0.65 (1.2)	0 (0)	− 100%	0.14 (10.6)	0.38 (21.7)	0.38 (24.4)	0.21 (4.7)	+50%	0.62 (0.6)	2.22 (2.1)	4.47 (4.1)	6.10 (5.6)	+884%
Sports drinks	0 (0)	0 (0)	0 (0)	0 (0)	-	0.07 (5.3)	0 (0)	0.01 (0.6)	0 (0)	− 100%	0.48 (0.4)	0.31 (0.3)	1.56 (1.4)	1.04 (1.0)	+117%
Yogurt	6.11 (10.7)	2.73 (6.6)	2.88 (5.5)	1.08 (4.6)	− 82%	0.04 (3.0)	0.19 (10.9)	0.01 (0.6)	0 (0)	− 100%	9.78 (8.7)	6.66 (6.2)	2.75 (2.5)	2.12 (2.0)	− 78%
Total	57.07 (100)	41.13 (100)	52.08 (100)	23.62 (100)	− 59%	1.32 (100)	1.75 (100)	1.56 (100)	4.45 (100)	+237%	111.8 (100)	107.6 (100)	107.9 (100)	108.3 (100)	− 3%

Child-appealing stations included Teletoon and YTV. Adolescent-appealing stations included Much Music and MTV. All other stations were classified as generalist stations. Data source: Nielsen Media Research (2011, 2013, 2016) and Numerator (2019).

In May 2019, 17.3% of children’s overall exposure to food advertising occurred while watching child-appealing stations (Table 5). The predominant source of exposure by station type varied among food categories. Most of children’s exposure to candy (76% of exposure), cereal (55%) and portable snack (59%) advertisements in May 2019 occurred on child-appealing stations. Conversely, most of children’s exposure to all other food categories, including regular soft drinks (100% of exposure), snack foods (97%), chocolate (96%), sit-down restaurants (93%) and fast food restaurants (92%), occurred on adolescent-appealing or generalist stations.

**Table 5:** Share of children’s exposure to food and beverage advertising viewed on child-appealing stations versus other stations in Toronto (Canada) in May 2019, overall and within food categories

Food/beverage category	Child-appealing stations (*n* = 2)		Other stations (*n* = 28)		Total exposure
	Avg number of ads viewed/child aged 2–11	% of total exposure within the food category	Avg number of ads viewed/child aged 2–11	% of total exposure within the food category	
Cakes	0.1	6.4	1.47	93.6	1.57
Candy	5.85	75.6	1.89	24.4	7.74
Cereal	8.24	55.6	6.57	44.4	14.81
Cheese	1.42	35.3	2.6	64.7	4.02
Chocolate	0.33	3.9	8.24	96.1	8.57
Compartment snacks	0	-	0	-	0.0
Cookies	0	0.0	0.7	100	0.7
Energy drinks	0.02	1.5	1.33	98.5	1.35
Ice cream	0.32	29.4	0.77	70.6	1.09
Juices,drinks and nectars	0.02	0.5	3.66	99.5	3.68
Pizza	0	0.0	1.08	100.0	1.08
Portable snacks	0.16	59.3	0.11	40.7	0.27
Restaurant - fast food	5.00	8.5	53.68	91.5	58.68
Restaurant - sit-down	0.80	6.7	11.14	93.3	11.94
Snack foods	0.28	2.9	9.53	97.1	9.81
Soft drinks - diet	0	0.0	0.54	100	0.54
Soft drinks - regular	0	0.0	6.31	100	6.31
Sports drinks	0	0.0	1.04	100	1.04
Yogurt	1.08	33.9	2.11	66.1	3.19
Total	23.6	17.3	112.8	82.7	136.4

Child-appealing stations included Teletoon and YTV. Data source: Nielsen Media Research (2011, 2013, 2016) and Numerator (2019).

## Discussion

Our study found that children in Toronto viewed on average 136 food ads in May 2019, 34 fewer than in May 2011. Despite this decline, one could conservatively estimate that children viewed at least 1700 food advertisements in 2019 on television alone, adding to and likely heightening the effects of other sources of food advertising exposure (Luxton et al., 2015; Norman et al., 2018). While we did not assess the healthfulness of promoted food products, clearly unhealthy food categories such as fast food, candy, chocolate, snack foods and regular soft drinks constituted more than half of children’s overall exposure in May 2019. Consistent with previous research, these findings further emphasize the failure of industry self-regulation in protecting Canadian children from exposure to unhealthy food advertising on television (Potvin Kent et al., 2011a; Kelly et al., 2019; Potvin Kent & Wanless, 2014; Potvin Kent et al., 2018).

Interestingly, children’s 20% decline in food advertising exposure occurred despite 22,000 additional food ads airing in May 2019 compared with May 2011 across examined stations. Shifts in exposure however varied by station category. Children’s exposure on child-appealing stations decreased by 59% even though ad frequency increased in equal measure (+59%). In contrast, children’s exposure on adolescent-appealing stations increased 237%, in tandem with a 145% increase in ad frequency, while remaining relatively constant on generalist stations, decreasing by only 3% despite an 18% increase in ads. As advertising exposure is dependent on both the volume and placement of advertisements as well as media consumption, children’s changing viewership behaviours are likely responsible for this decline. Children’s viewership of broadcast television did in fact decrease in recent years, going from an average of 22.2 h per week to 17.3 h per week between 2011–2012 and 2017–2018 (CRTC, 2020; CRTC, 2017). Children are likely consuming more television content, particularly children’s programming, via streaming platforms such as YouTube, Netflix and Disney+. As a result, one could expect their food advertising exposure from broadcast television to continue to decline over time. Although Netflix and Disney+ are free of traditional advertising, the fragmentation of television content across these multiplying platforms and the cost tied to their access may contribute to disparities in exposure between children of lower and higher income households. At the same time, children watching content on streaming platforms with advertising may be viewing more persuasive food promotions as metadata and behavioural targeting are being used to deliver highly personalized advertisements (World Health Organization, 2016). Despite the emergence of these newer platforms, broadcast television remains a significant source of advertising exposure that merits continued monitoring. Additional research is also needed to monitor advertising practices and exposure on streaming television.

Notably, our study found that 83% of advertisements viewed by children occurred on stations that appeal to older youth or are intended for general audiences, with the greatest absolute increases in exposure for some unhealthy food categories (e.g., snack foods) occurring on generalist stations. In some instances, decreases in advertisement frequency or exposure that occurred on child-appealing stations were offset by increases in advertising and exposure on generalist stations. Such was the case for fast food. Between May 2011 and May 2019, children’s exposure to fast food advertising decreased on average by 11 ads per child on child-appealing stations, where ad frequency remained fairly stable, while increasing on average by 14 ads per child on generalist stations, where the number of fast food advertisements increased by 60%. Similarly, even though regular soft drinks were not at all advertised on child-appealing stations in May 2019, ad exposure for these beverages reached its highest during the same period, with the near totality of this increase occurring on generalist stations where the number of soft drink ads was also at its highest. These findings illustrate that children’s exposure to unhealthy food advertising can increase even when such advertising remains stable or is absent from stations intended for young audiences.

Given that generalist stations accounted for a large share of children’s exposure, food advertising restrictions currently being considered by the federal government need to be designed such that children are adequately protected from unhealthy food advertising on programming that appeal to broad audiences. Based on policy evaluations from the United Kingdom, where unhealthy food advertising to children under 16 has been restricted on broadcast television since 2007, a recent World Health Organization report concluded that food advertising restrictions need to apply during programs that reach large absolute numbers of children, regardless of their intended audience, to be effective (Boyland et al., 2018). To increase the effectiveness of their restrictions, the UK is in fact expected to prohibit unhealthy food advertising airing before 9 pm on all broadcast television stations (Sweney, 2020). Canadian policymakers should consider heeding the lessons learned from abroad when crafting food advertising restrictions and ensure that Canadian children are also protected on television stations that appeal to broad audiences and not simply on child-appealing stations.

### Strengths and limitations

The limitations of our study are important to underscore. To start, our estimate of children’s exposure to food advertising is likely underestimated and may not be representative of children’s overall exposure on broadcast television. Our study only included advertisements for 19 food categories broadcast in Toronto on the 30 stations that were consistently recorded and monitored by Nielsen or Numerator across time. This led to the exclusion of the Disney Channel, a popular child-appealing station, for which data were only available for May 2019. As we only examined 4 weeks of advertising per year in one Canadian city, our estimate of exposure is likely not representative of all Canadian children and does not account for seasonal variations in advertising frequency and content (Pinto et al., 2020). Our study also excludes exposure to food product or brand appearances that can be seen during sponsored sporting events and other television programming (Elsey & Harris, 2016; Sherriff et al., 2010). While classifying advertisements into mutually exclusive food categories allowed us to test differences in the distribution of food ads across time, it did result in the underestimation of ad frequency and exposure of two food categories, namely portable snacks and diet soft drinks. Finally, given that advertising exposure data are only available in aggregate, statistical testing of differences across time and between station categories could not be performed. Future studies that measure children’s exposure objectively or through self-reporting should conduct multivariable analyses to investigate disparities in exposure among subgroups of interest (e.g., children from lower vs higher income households). Notwithstanding these limitations, our study provided recent estimates of food advertising exposure among Canadian children using measured media data and examined trends over time.

## Conclusion

Canadian children under 12 years of age are exposed to many food advertisements promoting unhealthy food categories on television and most of this exposure is occurring on generalist stations. Statutory restrictions currently being considered by Canadian policymakers should be designed such that children are adequately protected from unhealthy food advertising on both stations intended for general audiences and those targeting younger audiences.

### Contributions to knowledge

What does this study add to existing knowledge?

- This study provides recent estimates of children’s exposure to food advertising on broadcast television in a Canadian context and describes changes in exposure over time.
- Although children’s overall exposure to food advertising decreased between May 2011 and May 2019, their exposure remained high in 2019 and unhealthy food categories still accounted for most of this exposure.
- Most of children’s exposure to food advertising also stemmed from stations intended for general audiences, and their exposure to some unhealthy food categories (e.g., fast food and soft drinks) increased even when such advertising remained relatively stable or was absent from stations intended for young audiences.

What are the key implications for public health interventions, practice or policy?

- Industry self-regulation is not protecting Canadian children from exposure to unhealthy food advertising on broadcast television.
- Restricting the advertising of unhealthy food and beverages on child-appealing stations alone will not adequately protect them from such advertising.
- Statutory restrictions should be adopted and designed such that children are effectively protected from unhealthy food advertising on both stations intended for general audiences and those that appeal to younger audiences.

## Supplementary information

ESM 1 (DOCX 15 kb)

## Declarations

### Disclaimer

Funders had no role in designing the study, conducting the analyses, interpreting the findings and drafting the article nor the decision to publish.
