# Supplementary material for: Children’s measured exposure to food and beverage advertising on television in Toronto (Canada), May 2011–May 2019
Source: Can J Public Health. 2021 Jun 15;112(6):1008–19. doi: 10.17269/s41997-021-00528-1 (PMC8651920; doi:10.17269/s41997-021-00528-1)
Supplement: Supplementary file 1 — (DOCX 15 kb) [file 41997_2021_528_MOESM1_ESM.docx]

**Supplemental Table 1: Definition of Nielsen/Numerator food categories included in the study**

| **Food/beverage category** | **Definition** |
| --- | --- |
| Cakes | All cakes and puddings, including items that are ready to eat or require additional preparation (excludes frozen pastry and pie shells) |
| Candy | Confectionary made from sugar, water, flavoring and food coloring (excludes candy with chocolate) |
| Cereal | Ready-to-eat products marketed as breakfast food (excludes infant cereals and oatmeal) |
| Cheese | Cheese products in various formats, e.g., brick, string or slice (excludes cottage cheese) |
| Chocolate | Individually wrapped chocolate and candy bars (excludes boxed chocolate and candy with chocolate) |
| Compartment snacks | Pre-packaged products sold as snacks or meals that contain two or more foods stored in separate compartments (e.g. Lunchables) |
| Cookies | Small baked sweet biscuits |
| Energy drinks | Drink products that are primarily consumed for the purpose of boosting one’s mental and physical stimulation |
| Ice cream | Includes ice cream, frozen yogurt, sherbet, sorbet and frozen treats made from these foods |
| Juices, drinks and nectars | Sweetened and unsweetened juices and beverages that come in liquid, frozen, concentrated, and powdered forms (excludes water, milk and alternatives, tea and coffee drinks, cocktail mixers, and alcoholic beverages) |
| Pizza | Pizza not sold in restaurants |
| Portable snacks | Cereal bars or squares, protein bars and fruit bars or snacks |
| Snack foods | Savory snacks such as chips, pretzels, cheese puffs, nuts and meat-based snacks like jerky (excludes crackers) |
| Fast food restaurants | Foods sold at restaurants where ordering is conducted at a counter or drive-through, where menu boards are placed above the counter, and the table is cleaned up by the customer |
| Restaurants | Restaurants that serve prepared food and beverages that are ordered from a menu once seated and are consumed on the premises |
| Soft drinks (regular) | Any non-alcoholic carbonated drink |
| Soft drinks (diet) | Diet versions of soft drinks |
| Sports drinks | Drink products that are primarily consumed to rehydrate the body and replace electrolytes lost during physical activity |
| Yogurt | Yogurt in tub, tube, and drink form (excludes frozen yogurt) |

**Source:** Nielsen Media Research/Numerator.

This table has been published previously in the following article: Czoli, C. D., Pauzé, E., & Potvin Kent, M. (2020). Exposure to food and beverage advertising on television among Canadian adolescents, 2011 to 2016. *Nutrients*, *12*(2):428.

**Supplemental Table 2: List of stations by type**

|  | **Station name** |
| --- | --- |
| **Child-appealing stations** | YTV  Teletoon |
| **Adolescent-appealing stations** | Much (formerly Much Music)  MTV |
| **Generalist stations** | Bravo  CBC  CBC News Channel  CTV  CHCH  City TV  Country Music Television  The Comedy Network  CTV2  Discovery Channel  DTour (formerly TVTropolis)  The Food Network  Global Television Network  HGTV Canada  History Canada  ONL (formerly Outoor Life Network)  OMNI Television 1  OMNI Television 2  Sportsnet 360 (formerly The Score)  Showcase  Slice (formerly Life Network)  Space  Sportsnet Ontario  The Weather Network  W Network (formerly Women’s Television Network)  Vision Television |
| **Excluded stations** | M3 (formerly MuchMoreMusic and Much More; discontinued in Aug 2016)  TSN (discontinued in Aug 2014)  Sun TV (discontinued Aug 2011)  Fox (discontinued Sept 2014)  Gusto (started in Nov 2016)  CP24 (available for 2019 only)  Disney Channel (available for 2019 only)  TSN 2 (available for 2019 only)  TSN 4 (available for 2019 only) |
